# The Effectiveness of Microneedle Bipolar Fractional Radiofrequency in Treating Neck Wrinkles

**DOI:** 10.1111/jocd.70981

**Published:** 2026-06-30

**Authors:** Prapawan Chawvavanich, Ratchanee Vipanurat, Voragarn Annoptham

**Affiliations:** ^1^ Institute of Dermatology, Ministry of Public Health Bangkok Thailand

**Keywords:** microneedle radiofrequency, radiofrequency, wrinkles

## Abstract

**Introduction:**

Wrinkles, particularly in the neck area, are a prominent sign of aging. In recent years, there has been an increasing demand for neck wrinkle treatment. Previous studies have demonstrated that microneedle bipolar fractional radiofrequency is effective for facial wrinkles, with minimal side effects. This has led to interest in utilizing microneedle bipolar fractional radiofrequency for treating neck wrinkles as a potentially effective and safer option.

**Objective:**

To evaluate the effectiveness and safety of microneedle bipolar fractional radiofrequency in the treatment of neck wrinkles.

**Methods:**

This pilot study involved 25 subjects with neck wrinkles, classified at level II‐III on the Fitzpatrick wrinkle assessment scale, with scores of 4 or higher. Each participant received two treatments of microneedle bipolar fractional radiofrequency, spaced 12 weeks apart. The primary outcome measures included physician‐assessed photographic comparisons and evaluations using the Global Aesthetic Improvement Scale (GAIS). Secondary outcomes included patient satisfaction and the occurrence of side effects. Satisfaction was measured via a questionnaire at 12 and 24 weeks post‐treatment, with any adverse effects also being documented.

**Results:**

The study involved 25 participants, comprising 23 females and 2 males. After the first treatment (12 weeks post‐treatment), photographic evaluations and GAIS scores showed that 19 participants (76%) experienced mild improvement, while 6 participants (24%) exhibited significant improvement. Following the second treatment (24 weeks post‐treatment), 13 participants (52%) showed mild improvement, 11 participants (44%) demonstrated significant improvement, and 1 participant (4%) reported marked improvement. Participant satisfaction was notably high, with an average score of 8.64 ± 0.76 out of 10 at 12 weeks, rising to 9.08 ± 0.76 at 24 weeks. Reported side effects included transient erythema in 96% of participants (resolving within 24–48 h), pruritus in 28% (resolving within 1–3 days), and small scabs in 40% (resolving within 3–5 days). No serious adverse effects, such as hyperpigmentation, folliculitis, or scarring, were observed.

**Conclusion:**

Microneedle bipolar fractional radiofrequency is a highly effective and safe treatment for neck wrinkles.

## 1 Introduction

Wrinkles, particularly in the neck area, are a prominent sign of aging. Recently, there has been an increasing trend in patients seeking treatment for neck wrinkles, with not only older individuals but also younger patients experiencing this issue. Several treatments are available for neck wrinkles, including lasers, botulinum toxin A injections, dermal fillers, and surgical procedures. However, these approaches have been associated with various side effects. Fractional laser resurfacing was previously employed to address wrinkles and sagging skin around the neck, with effective results. However, reports indicated up to 25% incidence of postinflammatory hyperpigmentation, particularly in individuals with darker skin types, classified as Fitzpatrick skin types III and IV [1]. In response to these concerns, radiofrequency (RF) devices were developed to reduce risks and complications. Initially approved in 2002 for cosmetic use in treating periocular wrinkles, RF devices later received approval in 2004 for facial wrinkles and in 2006 for wrinkles in other areas of the body [2].

RF works by converting electrical energy into heat energy, which causes collagen beneath the skin to contract and stimulates new collagen production. Microneedle RF can release energy at temperatures between 65°C and 75°C, generating minimal heat in the epidermis. Insulated microneedle RF technology allows energy to be delivered directly to the target skin layer, causing less injury to surrounding tissues [2, 3, 4]. Previous studies have demonstrated the effectiveness and minimal side effects of microneedle bipolar fractional radiofrequency in treating facial wrinkles. While hyperpigmentation may occur, it is mild and usually resolves within a month [5]. Therefore, fractional RF technology offers patients an effective and safer treatment option.

## 2 Objective

To evaluate the effectiveness and safety of microneedle bipolar fractional radiofrequency in the treatment of neck wrinkles.

## 3 Methods

This pilot study was approved by the Human Research Ethics Committee of the Institute of Dermatology and included 25 male and female participants, all aged 40 and above, presenting with neck wrinkles classified at level II‐III on the Fitzpatrick wrinkle assessment scale with scores of 4 or higher. All participants were required to complete the full course of follow‐up throughout the study. Exclusion criteria included individuals who were pregnant or breastfeeding, those with underlying conditions such as thyroid disorders, epilepsy, or heat‐induced urticaria, as well as participants with abnormal blood clotting disorders or those taking anticoagulants. Individuals with a history of keloid scarring, those who had undergone neck skin treatments—including filler injections, botulinum toxin A, laser therapy, or skin‐tightening procedures—within 6 months prior to the study, or those using topical retinoids were also excluded. Additionally, participants with skin diseases in the treatment area, acute infections, cancers, autoimmune disorders, pacemakers, or connective and elastic tissue diseases were not eligible for the study.

Once consent was obtained, each participant's demographic data, including age, gender, and medical history, were collected. Physical examination followed, during which participant's skin color was assessed using the Fitzpatrick skin type scale. Neck wrinkles were graded on the Fitzpatrick Wrinkle Assessment Scale. Participants underwent neck photography at three time points: prior to treatment (week 0), at week 12 and week 24. Imaging was performed using the Vectra system and a Nikon D700 digital camera equipped with an AF‐S VR Micro‐Nikkor 105 mm f/2.8G IF‐ED lens. Prior to treatment, all participants received 5% Prilocaine/Lidocaine topical anesthesia applied to the neck area for 60 min. Participants received two treatments for neck wrinkles using microneedle bipolar fractional radiofrequency (Morpheus8) at weeks 0 and 12, with a 12‐week interval between sessions. The depth of penetration was set at 2–3 mm, with energy levels (fluence) ranging from 15 to 25 J/cm^2^, depending on the thickness of the skin in the treatment area. Following each session, participants received cold compresses for 15–30 min and were instructed to apply white petrolatum twice daily for 3 days.

Treatment outcomes for all participants will be evaluated at weeks 12 and 24 using photographic assessments based on the Global Aesthetic Improvement Score (GAIS). Three independent dermatologists, who were not involved in the study, conducted the evaluations. To ensure consistency, the evaluators first reviewed approximately 10–20 images from a separate sample group not included in the study to assess inter‐rater reliability. The Intraclass Correlation Coefficient (ICC) was calculated, with a minimum acceptable value of 0.8 required before proceeding with the actual assessments. In addition, participants completed a satisfaction questionnaire at weeks 12 and 24, assessing their perception of the treatment outcomes. Side effects, including burning sensation, erythema, rash, pruritus, scabs, hyperpigmentation, scarring, or any other adverse symptoms, were documented at all follow‐up stages throughout the study.

## 4 Results

**FIGURE 1:**
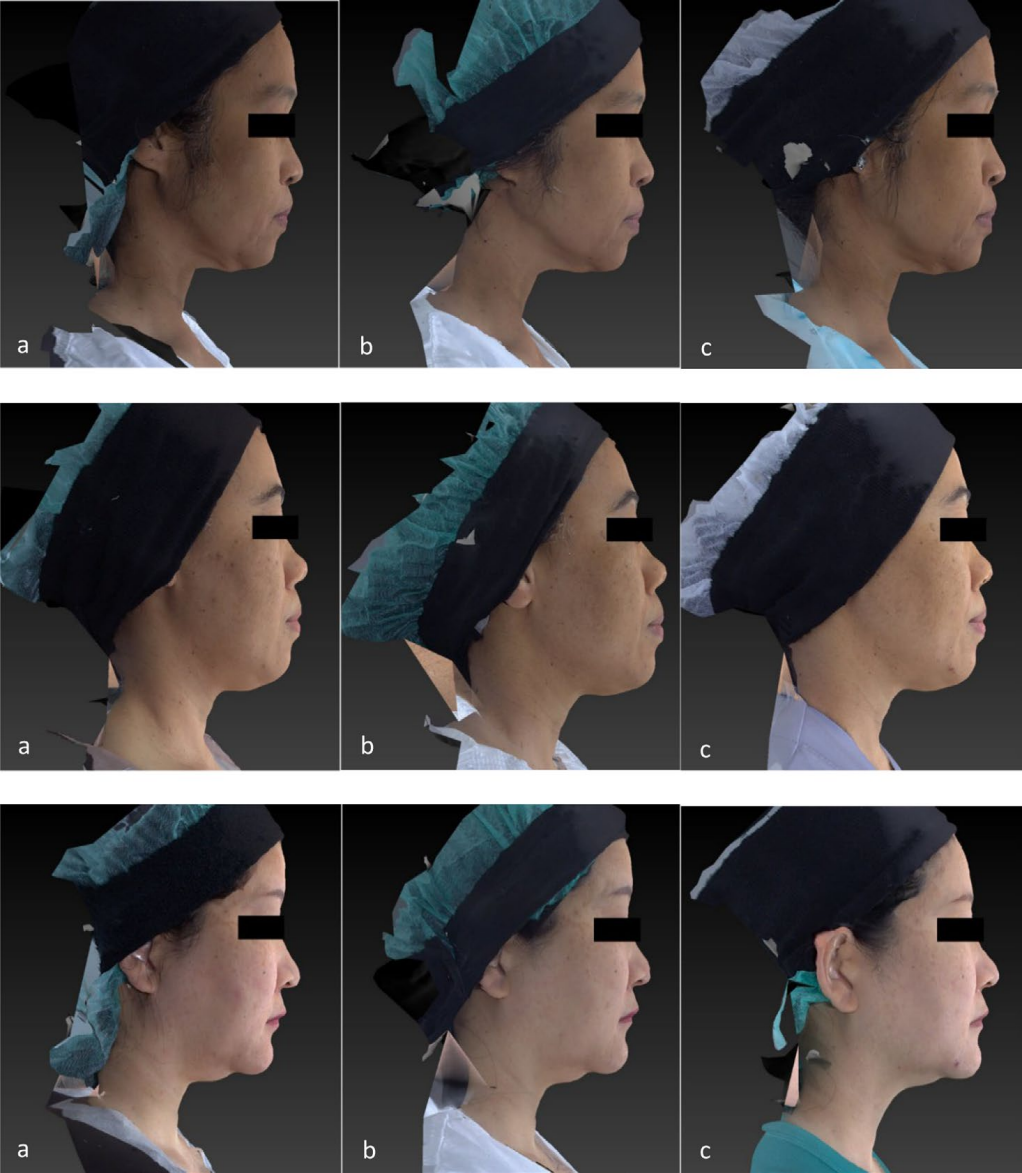
The representative patient photographs. (a) Before treatment. (b) 12 weeks after one treatment. (c) 12 weeks after two treatments.

**FIGURE 2:**
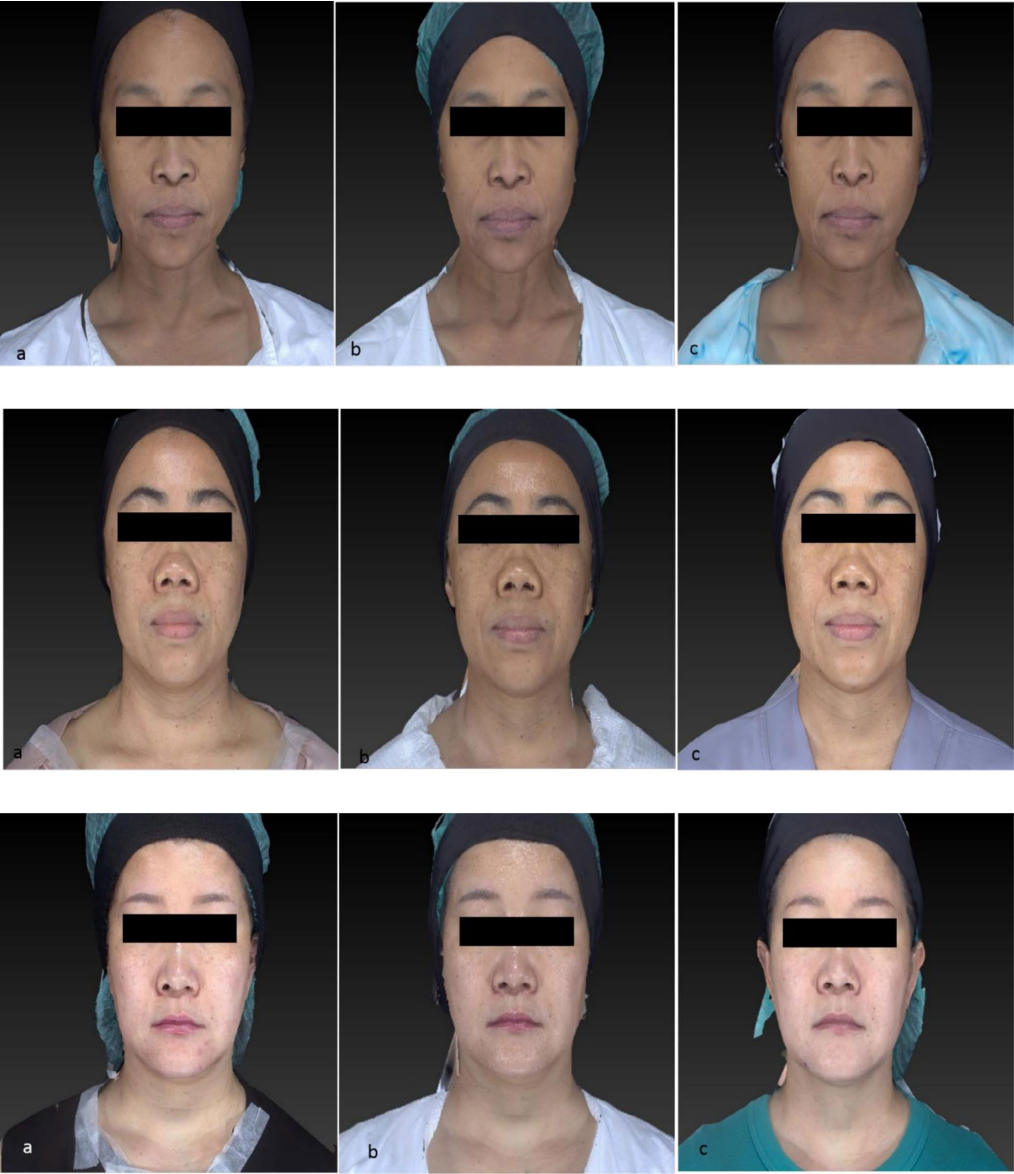
The representative patient photographs. (a) Before treatment. (b) 12 weeks after one treatment. (c) 12 weeks after two treatments.

The study comprised 25 participants, of which 23 (92%) were female and 2 (8%) were male. The participants ranged in age from 41 to 63 years, with a mean age of 51.56 ± 5.7 years. The majority of participants were classified as Fitzpatrick skin type III (19 participants, 76%), followed by type IV (5 participants, 20%) and type V (1 participant, 4%). All participants presented with neck wrinkles, graded using the Fitzpatrick Wrinkle Assessment Scale. 13 participants (52%) scored 4 points, 9 (36%) scored 5 points, and 3 (12%) scored 6 points, as detailed in Table 1. All participants completed the study and were evaluated using photographic analysis and the Global Aesthetic Improvement Scale (GAIS) by dermatologists. At the 12‐week follow‐up after the first treatment, all participants demonstrated improvement in their GAIS scores. 19 participants (76%) showed slight improvement, and 6 (24%) exhibited significant improvement in wrinkle reduction. By the 12‐week follow‐up after the second treatment, the percentage of participants with significant improvement had notably increased. 13 participants (52%) showed slight improvement, 11 (44%) demonstrated significant improvement, and 1 participant (4%) showed marked improvement as shown in Table 2. Inter‐rater reliability for the assessments was calculated, with a strong reliability score of 0.831, as presented in Table 3. Representative patient photographs before treatment, at 12 weeks, and at 24 weeks post‐treatment are shown in Figures 1 and 2. In addition, participant satisfaction with the treatment was high, with an average satisfaction score of 8.64 ± 0.76 out of 10 after the first treatment and 9.08 ± 0.76 after the second treatment. In terms of side effects, 96% of participants experienced transient erythema (resolving within 24–48 h), 28% reported pruritus (resolving within 1–3 days), and 40% had small scabs (resolving within 3–5 days). Notably, no participants developed post‐treatment hyperpigmentation or scarring (Table 4).

**TABLE 1:** Baseline characteristics of study participants.

	Results
Age (years)	41–63 years (The mean age is 51.56 ± 5.7 years)
Sex	
Male (Person)	2 (8%)
Female (Person)	23 (92%)
Fitzpatrick skin type	
Level 3 (Person)	19 (76%)
Level 4 (Person)	5 (20%)
Level 5 (Person)	1 (4%)
Fitzpatrick wrinkle assessment scale level II	25 (100%)
Fitzpatrick wrinkle score	
4 scores (Person)	13 (52%)
5 scores (Person)	9 (36%)
6 scores (Person)	3 (12%)

**TABLE 2:** The efficacy of skin quality restoration, as evaluated by physician 1, at three time points: Before treatment, 12 weeks post‐treatment, and 24 weeks post‐treatment.

	Week 12	Week 24
GAIS Level		
1 (no change)	0 (0%)	0 (0%)
2 (slight improvement)	19 (76%)	13 (52%)
3 (significant improvement)	6 (24%)	11 (44%)
4 (marked improvement)	0 (0%)	1 (4%)

**TABLE 3:** The inter‐observer reliability, measured by the consistency value between three assessors, was found to be 0.831 (95% CI: 0.730, 0.899).

GAIS	Week 12			Week 24		
Level	Physician 1	Physician 2	Physician 3	Physician 1	Physician 2	Physician 3
1	0	0	0	0	0	0
2	19	20	20	13	15	15
3	6	5	5	11	8	9
4	0	0	0	1	2	1

**TABLE 4:** Side effects after treatment.

Side effect	Participants (person)	%
Transient erythema (resolving within 24–48 h)	24	96
Pruritus (resolving within 1–3 days)	7	28
Small scabs (resolving within 3–5 days)	10	40
Hyperpigmentation	0	0
Scarring	0	0

## 5 Discussion

As individuals age, the structural integrity of the skin deteriorates across multiple layers, including the epidermis, dermis, and subcutaneous fat, along with the sagging of tendons. This degradation contributes to the formation of wrinkles and sagging skin. The neck area, in particular, is highly susceptible to these changes due to a lower density of sebaceous glands, fewer hair follicles, and a thinner dermal layer. Consequently, wound healing in the neck is slower and carries a higher risk of scarring and skin pigmentation irregularities, making it a particularly challenging area to treat. While neck lift surgery can address sagging skin, it does not restore the integrity of the epidermis and dermis. Furthermore, it requires an extended recovery period and poses a risk of postoperative scarring. The development of fractional laser resurfacing technology has shown efficacy in treating wrinkles in the neck region, targeting small areas of skin with energy, thereby minimizing damage to surrounding tissues and shortening recovery times. However, its impact on skin sagging is relatively low [6, 7]. Additionally, side effects, such as postinflammatory hyperpigmentation, have been reported in up to 25% of cases, particularly in individuals with darker skin tones (Fitzpatrick skin types III and IV) [1]. Botulinum toxin A injections can address platysmal bands, reduce the appearance of wrinkles and slight sagging, while also enhancing the definition of the jawline. However, the effects are temporary, typically lasting 3–6 months, requiring repeated treatments to maintain the desired results.

Radiofrequency (RF) technology converts electrical energy into thermal energy, leading to the contraction of collagen beneath the skin and stimulating the synthesis of new collagen, which results in firmer skin. The resistance of tissue to electrical current and the rate of heat generation vary among different tissue types. For instance, fat tissue acts as an insulator and possesses a high resistance to electrical current flow, generating significant heat. In contrast, water, which closely resembles a pure conductor, generates less heat. Optimal production of new collagen, elastin, and blood vessels occurs at temperatures between 65°C and 70°C. Additionally, the operation of RF waves is not dependent on chromophores, offering a safety advantage over laser treatments and making RF suitable for all skin types [2, 8]. The energy transmission mechanisms of RF devices are categorized into several types: monopolar RF, bipolar RF, and multipolar RF. Bipolar RF technology transmits energy between two adjacent electrodes, where energy flows from the positive electrode to the negative electrode. This configuration allows the energy to penetrate to a depth that is half the distance between the two poles, resulting in energy release in a shallower area of the skin compared to monopolar RF [2]. Radiofrequency technology has developed over time to make cosmetic treatments more effective. A recent advancement in technology is the development of RF microneedling techniques, which enable the controlled release of heat energy at targeted depths.

In this study, bipolar RF microneedle therapy was utilized to treat neck wrinkles. The device delivers energy via 24 insulated microneedles into the subcutaneous tissue. Energy is directed to the needle tip, targeting the specific skin layer for treatment. The dermis is heated to 65°C–70°C with minimal damage to the surrounding skin tissue and epidermis. The insulated needles limit dermal injury, leaving only a small puncture that heals within 24 h. Physicians can adjust the needle depth and energy settings as needed. For neck treatments, a penetration depth of 2–3 mm is applied, with fluence of 15–25 J/cm [8]. The study involved 25 participants with mild to moderate neck wrinkles (Fitzpatrick wrinkle assessment scale level II). All patients showed improvements in their Global Aesthetic Improvement Score (GAIS) after the first treatment (12 weeks post‐treatment), with 19 participants (76%) experiencing slight wrinkle improvement and 6 participants (24%) showing significant improvement. Following the second treatment (24 weeks after the first), 13 participants (52%) exhibited slight improvement, 11 (44%) showed significant improvement, and 1 participant (4%) achieved dramatic results. The study demonstrated that bipolar RF with insulated microneedles is highly effective, with efficacy improving as the number of treatments increases from one to two. The results remained strong 3 months following the final treatment. Moreover, all participants reported high satisfaction with the procedure, with an average satisfaction score of 8.64 ± 0.76 out of 10 after the first session, which increased to 9.08 ± 0.76 after the second session.

Previous studies on fractional CO2 lasers have reported adverse events such as erythema (lasting up to 14 days), swelling (for up to 3 days), crusting, and infection. Post‐inflammatory hyperpigmentation has been observed in up to 25% of patients with darker skin types (Fitzpatrick skin types III and IV) [1]. For botulinum toxin A injections to treat neck wrinkles, side effects include needle marks, bruising (8.9%), mild swallowing difficulty (5.2%), and neck muscle weakness (1.3%) [9]. Fractional radiofrequency has been associated with side effects like erythema, swelling (resolving within 2–3 days), bruising (resolving within 7–10 days), and hyperpigmentation in patients with darker skin types (resolving within 4 weeks) [8]. In this study, microneedle bipolar fractional radiofrequency was used for neck wrinkle treatment. The adverse effects observed included 96% erythema (resolving within 24–48 h), 28% pruritus (resolving within 1–3 days), and 40% small scabs (resolving within 3–5 days). Importantly, no serious side effects such as postinflammatory hyperpigmentation, folliculitis, infection, or scarring were reported.

However, the study had limitations, including a relatively small sample size, lack of long‐term follow‐up, absence of a comparative modality approach, and no instrumental measurements or quantitative evaluations.

## 6 Conclusions

In conclusion, microneedle bipolar fractional radiofrequency is a safe and effective treatment for neck wrinkles, offering a minimal recovery time. Several factors contribute to the formation of neck wrinkles and sagging, including undefined neck and jaw angles, loss of jawline definition, protrusion of submandibular fat, prominent platysmal bands, and skin laxity. For optimal results, treatments should be personalized to each individual's body and goals, and combining multiple therapeutic approaches can more effectively rejuvenate the neck's youthful appearance.

## Funding

This work was supported by the Institute of Dermatology, Ministry of Public Health, Bangkok, Thailand.

## Ethics Statement

This pilot study was approved by the Institutional Review Board of Dermatology Bangkok, Thailand. Study code: IRB/IEC 039/2564.

## Consent

All participants understood the study procedures and signed an informed consent form, including a photo consent form, before the study began.

## Conflicts of Interest

The authors declare no conflicts of interest.

## Data Availability

The data that support the findings of this study are available from the corresponding author upon reasonable request.

## References

[1] Y. Oram and A. D. Akkaya , “Neck Rejuvenation With Fractional CO_2_ Laser: Long‐Term Results,” Journal of Clinical and Aesthetic Dermatology 7 (2014): 23–29. 25161757 PMC4142817

[2] S. F. Weiner , “Radiofrequency Microneedling,” Facial Plastic Surgery Clinics of North America 27, no. 3 (2019): 291–303. 31280844 10.1016/j.fsc.2019.03.002

[3] M. T. Clementoni and G. S. Munavalli , “Fractional High Intensity Focused Radiofrequency in the Treatment of Mild to Moderate Laxity of the Lower Face and Neck: A Pilot Study,” Lasers in Surgery and Medicine 48, no. 5 (2016): 461–470. 26941115 10.1002/lsm.22499

[4] E. Dayan , C. Chia , A. J. Burns , and S. Theodorou , “Adjustable Depth Fractional Radiofrequency Combined With Bipolar Radiofrequency: A Minimally Invasive Combination Treatment for Skin Laxity,” Aesthetic Surgery Journal 39, no. Supplement_3 (2019): S112–S119. 30958550 10.1093/asj/sjz055 PMC6460431

[5] M. Alexiades‐Armenakas , J. Newman , A. Willey , et al., “Prospective Multicenter Clinical Trial of a Minimally Invasive Temperature‐Controlled Bipolar Fractional Radiofrequency System for Rhytid and Laxity Treatment,” Dermatologic Surgery 39, no. 2 (2013): 263–273. 23278964 10.1111/dsu.12065

[6] P. L. Bencini , A. Tourlaki , M. Galimberti , and G. Pellacani , “Non‐Ablative Fractionated Laser Skin Resurfacing for the Treatment of Aged Neck Skin,” Journal of Dermatological Treatment 9 (2014): 1–5. 10.3109/09546634.2014.933765 24953237

[7] E. P. Tierney and C. W. Hanke , “Ablative Fractionated CO_2_ Laser Resurfacing for the Neck: Prospective Study and Review of the Literature,” Journal of Drugs in Dermatology 8, no. 8 (2009): 723–731. 19663109

[8] I. A. Kleidona , D. Karypidis , N. Lowe , S. Myers , and A. Ghanem , “Fractional Radiofrequency in the Treatment of Skin Aging: An Evidence‐Based Treatment Protocol,” Journal of Cosmetic and Laser Therapy 22, no. 1 (2020): 9–25. 31825296 10.1080/14764172.2019.1674448

[9] C. M. Sugrue , J. L. Kelly , and N. McInerney , “Botulinum Toxin Treatment for Mild to Moderate Platysma Bands: A Systematic Review of Efficacy, Safety, and Injection Technique,” Aesthetic Surgery Journal 39, no. 2 (2019): 201–206. 30052764 10.1093/asj/sjy179

